# joineRML: a joint model and software package for time-to-event and multivariate longitudinal outcomes

**DOI:** 10.1186/s12874-018-0502-1

**Published:** 2018-06-07

**Authors:** Graeme L. Hickey, Pete Philipson, Andrea Jorgensen, Ruwanthi Kolamunnage-Dona

**Affiliations:** 1 Department of Biostatistics, Institute of Translational Medicine, University of Liverpool, Waterhouse Building, 1-5 Brownlow Street, Liverpool, L69 3GL UK; 2 Department of Mathematics, Physics and Electrical Engineering, Northumbria University, Ellison Place, Newcastle upon Tyne, NE1 8ST UK

**Keywords:** Joint modelling, Longitudinal data, Multivariate data, Time-to-event data, Software

## Abstract

**Background:**

Joint modelling of longitudinal and time-to-event outcomes has received considerable attention over recent years. Commensurate with this has been a rise in statistical software options for fitting these models. However, these tools have generally been limited to a single longitudinal outcome. Here, we describe the classical joint model to the case of *multiple* longitudinal outcomes, propose a practical algorithm for fitting the models, and demonstrate how to fit the models using a new package for the statistical software platform R, joineRML.

**Results:**

A multivariate linear mixed sub-model is specified for the longitudinal outcomes, and a Cox proportional hazards regression model with time-varying covariates is specified for the event time sub-model. The association between models is captured through a zero-mean multivariate latent Gaussian process. The models are fitted using a Monte Carlo Expectation-Maximisation algorithm, and inferences are based on approximate standard errors from the empirical profile information matrix, which are contrasted to an alternative bootstrap estimation approach. We illustrate the model and software on a real data example for patients with primary biliary cirrhosis with three repeatedly measured biomarkers.

**Conclusions:**

An open-source software package capable of fitting multivariate joint models is available. The underlying algorithm and source code makes use of several methods to increase computational speed.

**Electronic supplementary material:**

The online version of this article (10.1186/s12874-018-0502-1) contains supplementary material, which is available to authorized users.

## Background

In many clinical studies, subjects are followed-up repeatedly and response data collected. For example, routine blood tests might be performed at each follow-up clinic appointment for patients enrolled in a randomized drug trial, and biomarker measurements recorded. An event time is also usually of interest, for example time of death or study drop-out. It has been repeatedly shown elsewhere that if the longitudinal and event-time outcomes are correlated, then modelling the two outcome processes separately, for example using linear mixed models and Cox regression models, can lead to biased effect size estimates [1]. The same criticism has also been levelled at the application of so-called two-stage models [2]. The motivation for using joint models can be broadly separated into interest in drawing inference about (1) the time-to-event process whilst adjusting for the intermittently measured (and potentially error-prone) longitudinal outcomes, and (2) the longitudinal data process whilst adjusting for a potentially informative drop-out mechanism [3]. The literature on joint modelling is extensive, with excellent reviews given by Tsiatis and Davidian [4], Gould et al. [5], and the book by Rizopoulos [6].

Joint modelling has until recently been predominated by modelling a single longitudinal outcome together with a solitary event time outcome; herein referred to as *univariate joint modelling*. Commensurate with methodological research has been an increase in wide-ranging clinical applications (e.g. [7]). Recent innovations in the field of joint models have included the incorporation of multivariate longitudinal data [8], competing risks data [9, 10], recurrent events data [11], multivariate time-to-event data [12, 13], non-continuous repeated measurements (e.g. count, binary, ordinal, and censored data) [14], non-normally and non-parametrically distributed random effects [15], alternative estimation methodologies (e.g. Bayesian fitting and conditional estimating equations) [16, 17], and different association structures [18]. In this article, we specifically focus on the first innovation: multivariate longitudinal data. In this situation, we assume that multiple longitudinal outcomes are measured on each subject, which can be unbalanced and measured at different times for each subject.

Despite the inherently obvious benefits of harnessing all data in a single model or the published research on the topic of joint models for multivariate longitudinal data, a recent literature review by Hickey et al. [19] identified that publicly available software for fitting such models was lacking, which has translated into limited uptake by biomedical researchers. In this article we present the classical joint model described by Henderson et al. [3] extended to the case of multiple longitudinal outcomes. An algorithm proposed by Lin et al. [20] is used to fit the model, augmented by techniques to reduce the computational fitting time, including a quasi-Newton update approach, variance reduction method, and dynamic Monte Carlo updates. This algorithm is encoded into a R sofware package–joineRML. A simulation analysis and real-world data example are used to demonstrate the accuracy of the algorithm and the software, respectively.

## Implementation

As a prelude to the introduction and demonstration of the newly introduced software package, in the following section we describe the underlying model formulation and model fitting methodology.

### Model

For each subject *i*=1,…,*n*, $\boldsymbol {y}_{i} = \left (\boldsymbol {y}_{i1}^{\top }, \dots, \boldsymbol {y}_{iK}^{\top }\right)$ is the *K*-variate continuous outcome vector, where each ***y***_*ik*_ denotes an (*n*_*ik*_×1)-vector of observed longitudinal measurements for the *k*-th outcome type: $\boldsymbol {y}_{ik} = (y_{i1k}, \dots, y_{in_{ik}k})^{\top }$. Each outcome is measured at observed (possibly pre-specified) times *t*_*ijk*_ for *j*=1,…,*n*_*ik*_, which can differ between subjects and outcomes. Additionally, for each subject there is an event time $T_{i}^{*}$, which is subject to right censoring. Therefore, we observe $T_{i} = \min (T_{i}^{*}, C_{i})$, where *C*_*i*_ corresponds to a potential censoring time, and the failure indicator *δ*_*i*_, which is equal to 1 if the failure is observed $(T_{i}^{*} \leq C_{i})$ and 0 otherwise. We assume that both censoring and measurement times are non-informative.

The model we describe is the natural extension of the model proposed by Henderson et al. [3] to the case of multivariate longitudinal data. The model posits an unobserved or latent zero-mean (*K*+1)-variate Gaussian process that is realised independently for each subject, $W_{i}(t) = \left \{W_{1i}^{(1)}(t), \dots, W_{1i}^{(K)}(t), W_{2i}(t)\right \}$. This latent process subsequently links the separate sub-models via association parameters.

The *k*-th longitudinal data sub-model is given by

1 $$ y_{ik}(t) = \mu_{ik}(t) + W_{1i}^{(k)}(t) + \varepsilon_{ik}(t),  $$

where *μ*_*ik*_(*t*) is the mean response, and *ε*_*ik*_(*t*) is the model error term, which we assume to be independent and identically distributed normal with mean 0 and variance $\sigma _{k}^{2}$. The mean response is specified as a linear model

2 $$ \mu_{ik}(t) = \boldsymbol{x}_{ik}^{\top}(t) \boldsymbol{\beta}_{k},  $$

where ***x***_*ik*_(*t*) is a *p*_*k*_-vector of (possibly) time-varying covariates with corresponding fixed effect terms ***β***_*k*_. $W_{1i}^{(k)}(t)$ is specified as

3 $$ W_{1i}^{(k)}(t) = \boldsymbol{z}_{ik}^{\top}(t) \boldsymbol{b}_{ik},  $$

where ***z***_*ik*_(*t*) is an *r*_*k*_-vector of (possibly) time-varying covariates with corresponding subject-and-outcome random effect terms ***b***_*ik*_, which follow a zero-mean multivariate normal distribution with (*r*_*k*_×*r*_*k*_)-variance-covariance matrix ***D***_*kk*_. To account for dependence between the different longitudinal outcome outcomes, we let cov(***b***_*ik*_,***b***_*il*_)=***D***_*kl*_ for *k*≠*l*. Furthermore, we assume *ε*_*ik*_(*t*) and ***b***_*ik*_ are uncorrelated, and that the censoring times are independent of the random effects. These distributional assumptions together with the model given by (1)–(3) are equivalent to the multivariate extension of the Laird and Ware [21] linear mixed effects model. More flexible specifications of $W_{1i}^{(k)}(t)$ can be used [3], including for example, stationary Gaussian processes. However, we do not consider these cases here owing to the increased computational burden it carries, even for the univariate case.

The sub-model for the time-to-event outcome is given by the hazard model

$$ \lambda_{i}(t) = \lambda_{0}(t) \exp \left\{\boldsymbol{v}_{i}^{\top}(t) \boldsymbol{\gamma}_{v} + W_{2i}(t)\right\}, $$

where *λ*_0_(·) is an unspecified baseline hazard, and ***v***_*i*_(*t*) is a *q*-vector of (possibly) time-varying covariates with corresponding fixed effect terms ***γ***_*v*_. Conditional on *W*_*i*_(*t*) and the observed covariate data, the longitudinal and time-to-event data generating processes are conditionally independent. To establish a latent association, we specify *W*_2*i*_(*t*) as a linear combination of $\left \{W_{1i}^{(1)}(t), \dots, W_{1i}^{(K)}(t)\right \}$:

$$ W_{2i}(t) = \sum\limits_{k=1}^{K} \gamma_{yk} W_{1i}^{(k)}(t), $$

where ***γ***_*y*_=(*γ*_*y*1_,…,*γ*_*yK*_) are the corresponding association parameters. To emphasise the dependence of *W*_2*i*_(*t*) on the random effects, we explicitly write it as *W*_2*i*_(*t*,***b***_*i*_) from here onwards. As per $W_{1i}^{(k)}(t)$, *W*_2*i*_(*t*,***b***_*i*_) can also be flexibly extended, for example to include subject-specific frailty effects [3].

### Estimation

#### Likelihood

For each subject *i*, let $\boldsymbol {X}_{i} = \bigoplus _{k = 1}^{K} \boldsymbol {X}_{ik}$ and $\boldsymbol {Z}_{i} = \bigoplus _{k = 1}^{K} \boldsymbol {Z}_{ik}$ be block-diagonal matrices, where $\boldsymbol {X}_{ik} = \left (\boldsymbol {x}_{i1k}^{\top }, \dots, \boldsymbol {x}_{in_{ik}k}^{\top }\right)$ is an (*n*_*ik*_×*p*_*k*_)-design matrix, with the *j*-th row corresponding to the *p*_*k*_-vector of covariates measured at time *t*_*ijk*_, and $\bigoplus $ denotes the direct matrix sum. The notation similarly follows for the random effects design matrices, ***Z***_*ik*_. We denote the error terms by a diagonal matrix $\boldsymbol {\Sigma }_{i} = \bigoplus _{k = 1}^{K} \sigma _{k}^{2} \boldsymbol {I}_{n_{ik}}$ and write the overall variance-covariance matrix for the random effects as

$$ \boldsymbol{D} = \left(\begin{array}{ccc} \boldsymbol{D}_{11} & \cdots & \boldsymbol{D}_{1K} \\ \vdots & \ddots & \vdots \\ \boldsymbol{D}_{1K}^{\top} & \cdots & \boldsymbol{D}_{KK} \\ \end{array}\right), $$

where ***I***_*n*_ denotes an *n*×*n* identity matrix. We further define $\boldsymbol {\beta } = \left (\boldsymbol {\beta }_{1}^{\top }, \dots, \boldsymbol {\beta }_{K}^{\top }\right)^{\top }$ and $\boldsymbol {b}_{i} = \left (\boldsymbol {b}_{i1}^{\top }, \dots, \boldsymbol {b}_{iK}^{\top }\right)^{\top }$. Hence, we can then rewrite the longitudinal outcome sub-model as

$$\begin{array}{@{}rcl@{}} \boldsymbol{y}_{i} \,|\, \boldsymbol{b}_{i}, \boldsymbol{\beta}, \boldsymbol{\Sigma}_{i} &\sim& N(\boldsymbol{X}_{i} \boldsymbol{\beta} + \boldsymbol{Z}_{i} \boldsymbol{b}_{i}, \boldsymbol{\Sigma}_{i}), \\ \text{with}\ \boldsymbol{b}_{i} \,|\, \boldsymbol{D} &\sim& N(\boldsymbol{0}, \boldsymbol{D}). \end{array} $$

For the estimation, we will assume that the covariates in the time-to-event sub-model are time-independent and known at baseline, i.e. ***v***_*i*_≡***v***_*i*_(0). Extensions of the estimation procedure for time-varying covariates are outlined elsewhere [6, p. 115]. The *observed* data likelihood for the joint outcome is given by

4 $$ \prod\limits_{i=1}^{n} \left(\int_{-\infty}^{\infty} f(\boldsymbol{y}_{i} \,|\, \boldsymbol{b}_{i}, \boldsymbol{\theta}) f(T_{i}, \delta_{i} \,|\, \boldsymbol{b}_{i}, \boldsymbol{\theta}) f(\boldsymbol{b}_{i} \,|\, \boldsymbol{\theta}) d\boldsymbol{b}_{i} \right),  $$

where $\boldsymbol {\theta } = \left (\boldsymbol {\beta }^{\top }, \text {vech}(\boldsymbol {D}), \sigma _{1}^{2}, \dots, \sigma _{K}^{2}, \lambda _{0}(t), \boldsymbol {\gamma }_{v}^{\top }, \boldsymbol {\gamma }_{y}^{\top }\right)$ is the collection of unknown parameters that we want to estimate, with vech(***D***) denoting the half-vectorisation operator that returns the vector of lower-triangular elements of matrix ***D***.

As noted by Henderson et al. [3], the observed data likelihood can be calculated by rewriting it as

$$ \prod\limits_{i=1}^{n} f(\boldsymbol{y}_{i} \,|\, \boldsymbol{\theta}) \left(\int_{-\infty}^{\infty} f(T_{i}, \delta_{i} \,|\, \boldsymbol{b}_{i}, \boldsymbol{\theta}) f(\boldsymbol{b}_{i} \,|\, \boldsymbol{y}_{i}, \boldsymbol{\theta}) d\boldsymbol{b}_{i} \right), $$

where the marginal distribution *f*(***y***_*i*_ | ***θ***) is a multivariate normal density with mean ***X***_*i*_***β*** and variance-covariance matrix $\boldsymbol {\Sigma }_{i} + \boldsymbol {Z}_{i} \boldsymbol {D} \boldsymbol {Z}_{i}^{\top }$, and *f*(***b***_*i*_ | ***y***_*i*_,***θ***) is given by (6).

#### MCEM algorithm

We determine maximum likelihood estimates of the parameters ***θ*** using the Monte Carlo Expectation Maximisation (MCEM) algorithm [22], by treating the random effects ***b***_*i*_ as missing data. This is effectively the same as the conventional Expectation-Maximisation (EM) algorithm, as used by Wulfsohn and Tsiatis [23] and Ratcliffe et al. [24] in the context of fitting univariate data joint models, except the E-step exploits a Monte Carlo (MC) integration routine as opposed to Gaussian quadrature methods, which we expect to be beneficial when the dimension of random effects becomes large.

Starting from an initial estimate of the parameters, $\hat {\boldsymbol {\theta }}^{(0)}$, the procedure involves iterating between the following two steps until convergence is achieved.

1. *E-step*. At the (*m*+1)-th iteration, we compute the expected log-likelihood of the *complete* data conditional on the *observed* data and the current estimate of the parameters,
  $$\begin{array}{@{}rcl@{}} {\selectfont{\begin{aligned} {} Q(\boldsymbol{\theta} \,|\, \hat{\boldsymbol{\theta}}^{(m)}) &= \sum\limits_{i=1}^{n} \mathbb{E} \Big\{\log f(\boldsymbol{y}_{i}, T_{i}, \delta_{i}, \boldsymbol{b}_{i} \,|\, \boldsymbol{\theta})\Big\} \\ &= \sum\limits _{i=1}^{n} \int_{-\infty}^{\infty} \Big\{\log f(\boldsymbol{y}_{i}, T_{i}, \delta_{i}, \boldsymbol{b}_{i} \,|\, \boldsymbol{\theta})\Big\} f(\boldsymbol{b}_{i} \,\!|\, T_{i}, \delta_{i}, \boldsymbol{y}_{i};\hat{\boldsymbol{\theta}}^{(m)}) d\boldsymbol{b}_{i}. \end{aligned}}} \end{array} $$
  Here, the complete-data likelihood contribution for subject *i* is given by the integrand of (4).
2. *M-step*. We maximise $Q(\boldsymbol {\theta } \,|\, \hat {\boldsymbol {\theta }}^{(m)})$ with respect to ***θ***. Namely, we set
  $$ \hat{\boldsymbol{\theta}}^{(m+1)} = \underset{\boldsymbol{\theta}}{\text{argmax}}\ Q\left(\boldsymbol{\theta} \,|\, \hat{\boldsymbol{\theta}}^{(m)}\right). $$

The M-step estimators naturally follow from Wulfsohn and Tsiatis [23] and Lin et al. [20]. Maximizers for all parameters except ***γ***_*v*_ and ***γ***_*y*_ are available in closed-form; algebraic details are presented in Additional file 1. The parameters $\boldsymbol {\gamma } = (\boldsymbol {\gamma _{v}}^{\top }, \boldsymbol {\gamma }_{y}^{\top })^{\top }$ are jointly updated using a one-step Newton-Raphson algorithm as

$$ \hat{\boldsymbol{\gamma}}^{(m+1)} = \hat{\boldsymbol{\gamma}}^{(m)} + I\left(\hat{\boldsymbol{\gamma}}^{(m)}\right)^{-1} S\left(\hat{\boldsymbol{\gamma}}^{(m)}\right),$$

where $\hat {\boldsymbol {\gamma }}^{(m)}$ denotes the value of ***γ*** at the current iteration, $S\left (\hat {\boldsymbol {\gamma }}^{(m)}\right)$ is the corresponding score, and $I\left (\hat {\boldsymbol {\gamma }}^{(m)}\right)$ is the observed information matrix, which is equal to the derivative of the negative score. Further details of this update are given in Additional file 1. The M-step for ***γ*** is computationally expensive to evaluate. Therefore, we also propose a quasi-Newton one-step update by approximating $I\left (\hat {\boldsymbol {\gamma }}^{(m)}\right)$ by an empirical information matrix for ***γ***, which can be considered an analogue of the Gauss-Newton method [25, p. 8]. To further compensate for this approximation, we also use a nominal step-size of 0.5 rather than 1, which is used when using the Newton-Raphson update.

The M-step involves terms of the form $\mathbb {E}\left [h(\boldsymbol {b}_{i}) \,|\, T_{i},{\vphantom {\hat {\boldsymbol {\theta }}}}\right. \left.\delta _{i}, \boldsymbol {y}_{i}; \hat {\boldsymbol {\theta }}\right ]$, for known functions *h*(·). The conditional expectation of a function of the random effects can be written as

5 $$ {\selectfont{\begin{aligned} {} \mathbb{E}\left[h(\boldsymbol{b}_{i}) \,|\, T_{i}, \delta_{i}, \boldsymbol{y}_{i}; \hat{\boldsymbol{\theta}}\right] = \frac{\int_{-\infty}^{\infty} h(\boldsymbol{b}_{i}) f(\boldsymbol{b}_{i} \,|\, \boldsymbol{y}_{i}; \hat{\boldsymbol{\theta}}) f(T_{i}, \delta_{i} \,|\, \boldsymbol{b}_{i}; \hat{\boldsymbol{\theta}}) d\boldsymbol{b}_{i}}{\int_{-\infty}^{\infty} f(\boldsymbol{b}_{i} \,|\, \boldsymbol{y}_{i}; \hat{\boldsymbol{\theta}}) f(T_{i}, \delta_{i} \,|\, \boldsymbol{b}_{i}; \hat{\boldsymbol{\theta}}) d\boldsymbol{b}_{i}}, \end{aligned}}}  $$

where $f(T_{i}, \delta _{i} \,|\, \boldsymbol {b}_{i}; \hat {\boldsymbol {\theta }})$ is given by

$${\selectfont{\begin{aligned} {} f(T_{i}, \delta_{i} \,|\, \boldsymbol{b}_{i}; \boldsymbol{\theta}) = &\left[\lambda_{0}(T_{i}) \exp\left\{\boldsymbol{v}_{i}^{\top} \boldsymbol{\gamma}_{v} + W_{2i}(T_{i}, \boldsymbol{b}_{i})\right\}\right]^{\delta_{i}} \\ &\times \exp\left\{-\int_{0}^{T_{i}}\lambda_{0}(u)\exp\left\{\boldsymbol{v}_{i}^{\top} \boldsymbol{\gamma}_{v} + W_{2i}(u, \boldsymbol{b}_{i})\right\}du\right\} \end{aligned}}} $$

and $f(\boldsymbol {b}_{i} \,|\, \boldsymbol {y}_{i}; \hat {\boldsymbol {\theta }})$ is calculated from multivariate normal distribution theory as

6 $$ \boldsymbol{b}_{i} \,|\, \boldsymbol{y}_{i}, \boldsymbol{\theta} \sim N\left(\boldsymbol{A}_{i} \left\{\boldsymbol{Z}_{i}^{\top} \boldsymbol{\Sigma}_{i}^{-1}(\boldsymbol{y}_{i} - \boldsymbol{X}_{i} \boldsymbol{\beta})\right\}, \boldsymbol{A}_{i}\right),  $$

with $\boldsymbol {A}_{i} = \left (\boldsymbol {Z}_{i}^{\top } \boldsymbol {\Sigma }_{i}^{-1} \boldsymbol {Z}_{i} + \boldsymbol {D}^{-1}\right)^{-1}$. As this becomes computationally expensive using Gaussian quadrature commensurate with increasing dimension of ***b***_*i*_, we estimate the integrals by MC sampling such that the expectation is approximated by the ratio of the sample means for $h(\boldsymbol {b}_{i}) f(T_{i}, \delta _{i} \,|\, \boldsymbol {b}_{i}; \hat {\boldsymbol {\theta }})$ and $f(T_{i}, \delta _{i} \,|\, \boldsymbol {b}_{i}; \hat {\boldsymbol {\theta }})$ evaluated at each MC draw. Furthermore, we use antithetic simulation for variance reduction in the MC integration. Instead of directly sampling from (6), we sample ***Ω***∼*N*(0,***I***_*r*_) and obtain the *pairs*

$$ \boldsymbol{A}_{i} \left\{ \boldsymbol{Z}_{i}^{\top} \boldsymbol{\Sigma}_{i}^{-1} (\boldsymbol{y}_{i} - \boldsymbol{X}_{i} \boldsymbol{\beta}) \right\} \pm \boldsymbol{C}_{i} \boldsymbol{\Omega}, $$

where ***C***_*i*_ is the Cholesky decomposition of ***A***_*i*_ such that $\boldsymbol {C}_{i} \boldsymbol {C}_{i}^{\top } = \boldsymbol {A}_{i}$. Therefore we only need to draw *N*/2 samples using this approach, and by virtue of the negative correlation between the pairs, it leads to a smaller variance in the sample means taken in the approximation than would be obtained from *N* independent simulations. The choice of *N* is described below.

#### Initial values

The EM algorithm requires that initial parameters are specified, namely $\hat {\boldsymbol {\theta }}^{(0)}$. By choosing values close to the maximizer, the number of iterations required to reach convergence should be reduced.

For the time-to-event sub-model, a quasi-two-stage model is fitted when the measurement times are balanced, i.e. when *t*_*ijk*_=*t*_*ij*_∀*k*. That is, we fit *separate* LMMs for each longitudinal outcome as per (1), ignoring the correlation between different outcomes. This is straightforward to implement using standard software, in particular using lme() and coxph() from the R packages nlme [26] and survival [27], respectively. From the fitted models, the best linear unbiased predictions (BLUPs) of the separate model random effects are used to estimate each $W_{1i}^{(k)}(t)$ function. These estimates are then included as time-varying covariates in a Cox regression model, alongside any other fixed effect covariates, which can be straightforwardly fitted using standard software. In the situation that the data are not balanced, i.e. when *t*_*ijk*_≠*t*_*ij*_∀*k*, then we fit a standard Cox proportional hazards regression model to estimate ***γ***_*v*_ and set *γ*_*yk*_=0∀*k*.

For the longitudinal data sub-model, when *K*>1 we first find the maximum likelihood estimate of $\left \{\boldsymbol {\beta }, \text {vech}(\boldsymbol {D}), \sigma _{1}^{2}, \dots, \sigma _{K}^{2}\right \}$ by running a separate EM algorithm for the multivariate linear mixed model. Both the E- and M-step updates are available in closed form, and the initial parameters for this EM algorithm are available from the separate LMM fits, with ***D*** initialized as block-diagonal. As these are estimated using an EM rather than MCEM algorithm, we can specify a stricter convergence criterion on the estimates.

#### Convergence and stopping rules

Two standard stopping rules for the deterministic EM algorithm used to declare convergence are the relative and absolute differences, defined as

7 $$\begin{array}{@{}rcl@{}} \Delta_{\text{rel}}^{(m+1)} &=& \max\left\{\frac{\left|\hat{\boldsymbol{\theta}}^{(m+1)} - \hat{\boldsymbol{\theta}}^{(m)}\right|}{\left|\hat{\boldsymbol{\theta}}^{(m)}\right| + \epsilon_{1}}\right\} < \epsilon_{0}, \text{ and}  \end{array} $$

8 $$\begin{array}{@{}rcl@{}} \Delta_{\text{abs}}^{(m+1)} &=& \max\left\{\left|\hat{\boldsymbol{\theta}}^{(m+1)} - \hat{\boldsymbol{\theta}}^{(m)}\right|\right\} < \epsilon_{2}  \end{array} $$

respectively, for some appropriate choice of *ε*_0_, *ε*_1_, and *ε*_2_, where the maximum is taken over the components of ***θ***. For reference, the R package JM [28] implements (7) (in combination with another rule based on relative change in the likelihood), whereas the R package joineR [29] implements (8). The relative difference might be unstable about parameters near zero that are subject to MC error. Therefore, the convergence criterion for each parameter might be chosen separately at each EM iteration based on whether the absolute magnitude is below or above some threshold. A similar approach is adopted in the EM algorithms employed by the software package SAS [30, p. 330].

The choice of *N* and the monitoring of convergence are conflated when applying a MCEM algorithm, and a dynamic approach is required. As noted by [22], it is computationally inefficient to use a large *N* in the early phase of the algorithm when the parameter estimates are likely to be far from the maximizer. On the flip side, as the parameter estimates approach the maximizer, the stopping rules will fail as the changes in parameter estimates will be swamped by MC error. Therefore, it has been recommended that one increase *N* as the estimate moves towards the maximizer. Although this might be done subjectively [31] or by pre-specified rules [32], an automated approach is preferable and necessary for a software implementation. Booth and Hobert [33] proposed an update rule based on a confidence ellipsoid for the maximizer at the (*m*+1)-th iteration, calculated using an approximate sandwich estimator for the maximizer, which accounts for the MC error at each iteration. This approach requires additional variance estimation at each iteration, therefore we opt for a simpler approach described by Ripatti et al. [34]. Namely, we calculate a coefficient of variation at the (*m*+1)-th iteration as

$$ \text{cv}\left(\Delta_{\text{rel}}^{(m+1)}\right) = \frac{\text{sd}\left(\Delta_{\text{rel}}^{(m-1)}, \Delta_{\text{rel}}^{(m)}, \Delta_{\text{rel}}^{(m+1)}\right)}{\text{mean}\left(\Delta_{\text{rel}}^{(m-1)}, \Delta_{\text{rel}}^{(m)}, \Delta_{\text{rel}}^{(m+1)}\right)}, $$

where $\Delta _{\text {rel}}^{(m+1)}$ is given by (7), and sd(·) and mean(·) are the sample standard deviation and mean functions, respectively. If $\text {cv}\left (\Delta _{\text {rel}}^{(m+1)}\right) > \text {cv}\left (\Delta _{\text {rel}}^{(m)}\right)$, then *N*:=*N*+⌊*N*/*δ*⌋, for some small positive integer *δ*. Typically, we run the MCEM algorithm with a small *N* (for a fixed number of iterations—a *burn-in*) before implementing this update rule in order to get into the approximately correct parameter region. Appropriate values for other parameters will be application specific, however we have found *δ*=3, *N*=100*K* (for 100*K* burn-in iterations), *ε*_1_=0.001, and *ε*_0_=*ε*_2_=0.005 delivers reasonably accurate estimates in many cases, where *K* was earlier defined as the number of longitudinal outcomes.

As the EM monotonicity property is lost due to the MC integrations in the MCEM algorithm, convergence might be prematurely declared due to stochasticity if the *ε*-values are too large. To reduce the chance of this occurring, we require that the stopping rule is satisfied for 3 consecutive iterations [33, 34]. However, in any case, trace plots should be inspected to confirm convergence is appropriate.

#### Standard error estimation

Standard error (SE) estimation is usually based on inverting the observed information matrix. When the baseline hazard is unspecified, as is the case here, this presents several challenges. First, $\hat {\lambda }_{0}(t)$ will generally be a high-dimensional vector, which might lead to numerical difficulties in the inversion of the observed information matrix [6]. Second, the profile likelihood estimates based on the usual observed information matrix approach are known to be underestimated [35]. The reason for this is that the profile estimates are implicit, since the posterior expectations, given by (5), depend on the parameters being estimated, including *λ*_0_(*t*) [6, p. 67].

To overcome these challenges, Hsieh et al. [35] recommended to use bootstrap methods to calculate the SEs. However, this approach is computationally expensive. Moreover, despite the purported theoretical advantages, we also note that recently it has been suggested that bootstrap estimators might actually *overestimate* the SEs; e.g. [36, p. 740] and [35, p. 1041]. At the model development stage, it is often of interest to gauge the strength of association of model covariates, which is not feasible with repeated bootstrap implementations. Hence, an approximate SE estimator is desirable. In either case, the theoretical properties will be contaminated by the addition of MC error from the MCEM algorithm, and it is not yet fully understood what the ramifications of this are. Hence, any standard errors must be interpreted with a degree of caution. We consider two estimators below.

**1. Bootstrap method.** These are estimated by sampling *n* subjects with replacement and re-labelling the subjects with indices *i*^′^=1,…,*n*. We then re-fit the model to the bootstrap-sampled dataset. It is important to note that we re-sample subjects, not individual data points. This is repeated *B*-times, for a sufficiently large integer *B*. Since we already have the MLEs from the fitted model, we can use these as initial values for each bootstrap model fit, thus reducing initial computational overheads in calculating approximate initial parameters. For each iteration, we extract the model parameter estimates for $\left (\boldsymbol {\beta }^{\top }, \text {vech}(\boldsymbol {D}), \sigma _{1}^{2}, \dots, \sigma _{K}^{2}, \boldsymbol {\gamma }_{v}^{\top }, \boldsymbol {\gamma }_{y}^{\top }\right)$. Note that we do not estimate SEs for *λ*_0_(*t*) using this approach. However, they are generally not of inferential interest. When *B* is sufficiently large, the SEs can be estimated from the estimated coefficients of the bootstrap samples. Alternatively, 100(1−*α*)%-confidence intervals can be estimated from the the 100*α*/2-th and 100(1−*α*/2)-th percentiles.

**2. Empirical information matrix method.** Using the Breslow estimator for $\int _{0}^{t} \lambda _{0}(u) \mathrm {d}u$, the profile score vector for $\boldsymbol {\theta }_{-\lambda } = (\boldsymbol {\beta }^{\top }, \text {vech}(\boldsymbol {D}), \sigma _{1}^{2}, \dots, \sigma _{K}^{2}, \boldsymbol {\gamma }^{\top })$ is calculated (see Additional file 1). We approximate the profile information for ***θ***_−*λ*_ by $I_{e}^{-1/2}(\hat {\boldsymbol {\theta }}_{-\lambda _{0}})$, where $I_{e}(\boldsymbol {\theta }_{-\lambda _{0}})\phantom {\dot {i}\!}$ is the observed empirical information [25] given by

9 $$ I_{e}(\boldsymbol{\theta}_{-\lambda}) = \sum\limits_{i=1}^{n} s_{i}(\boldsymbol{\theta}_{-\lambda})^{\otimes 2} - \frac{1}{n} S(\boldsymbol{\theta}_{-\lambda})^{\otimes 2},  $$

*s*_*i*_(***θ***_−*λ*_) is the conditional expectation of the complete-data profile score for subject *i*, *S*(***θ***_−*λ*_) is the score defined by $S(\boldsymbol {\theta }_{-\lambda }) = \sum _{i=1}^{n} s_{i}(\boldsymbol {\theta }_{-\lambda })$, and ***a***^⊗2^=***aa***^⊤^ is outer product for a vector ***a***. At the maximizer, $S(\hat {\boldsymbol {\theta }}) = 0$, meaning that the right hand-side of (9) is zero. Due to the MC error in the MCEM algorithm, this will not be exactly zero, and therefore we include it in the calculations. As per the bootstrap approach, SEs for the baseline hazard are again not calculated. We note that this SE estimator will be subject to the exact same theoretical limitation of underestimation described by Hsieh et al. [35], since the profiling was implicit; that is, because the posterior expectations involve the parameters *θ*.

### Software

The model described here is implemented in the R package joineRML, which is available on the The Comprehensive R Archive Network (CRAN) (https://CRAN.R-project.org/package=joineRML). The principal function in joineRML is mjoint(). The primary arguments for implementing mjoint() are summarised in Table 1. To achieve computationally efficiency, parts of the MCEM algorithm in joineRML are coded in C++ using the Armadillo linear algebra library and integrated using the R package RcppArmadillo [37].

**Table 1:** The primary arguments^a^ with descriptions for the mjoint() function in the R package joineRML

Argument	Description
formLongFixed	a list of formulae for the fixed effects component of each longitudinal outcome. The left hand-hand side defines the response, and the right-hand side specifies the fixed effect terms.
formLongRandom	a list of one-sided formulae specifying the model for the random effects effects of each longitudinal outcome.
formSurv	a formula specifying the proportional hazards regression model (not including the latent association structure).
data	a list of data.frame objects for each longitudinal outcome in which to interpret the variables named in the formLongFixed and formLongRandom. The list structure enables one to include multiple longitudinal outcomes with different measurement protocols. If the multiple longitudinal outcomes are measured at the same time points for each patient (i.e. *t*_*ijk*_=*t*_*ij*_∀*k*), then a single data.frame object can be given instead of a list. It is assumed that each data frame is in *long format*.
survData	(optional) a data.frame in which to interpret the variables named in the formSurv. If survData is not given, then mjoint() looks for the time-to-event data in data.
timeVar	a character string indicating the time variable in the linear mixed effects model.
inits	(optional) a list of initial values for some or all of the parameters estimated in the model.
control	(optional) a list of control parameters. These allow for the control of *ε*_0_, *ε*_1_, and *ε*_2_ in (7) and (8); the choice of *N*, *δ*, and convergence criteria; the maximum number of MCEM iterations, and the minimum number of MCEM iterations during burn-in. Additionally, the control argument gammaOpt can be used to specify whether a one-step Newton-Raphson (=~NR~) or Gauss-Newton-like (=~GN~) update should be used for the M-step update of ***γ***.

^a^mjoint() also takes the optional additional arguments verbose, which if TRUE allows for monitoring updates at each MCEM algorithm iteration, and pfs, which if FALSE can force the function not to calculate post-fit statistics such as the BLUPs and associated standard errors of the random effects and approximate standard errors of the model parameters. In general, these arguments are not required

A model fitted using the mjoint() function returns an object of class mjoint. By default, approximate SE estimates are calculated using the empirical information matrix. If one wishes to use bootstrap standard error estimates, then the user can pass the model object to the bootSE() function. Several generic functions (or rather, S3 methods) can also be applied to mjoint objects, as described in Table 2. These generic functions include common methods, for example coef(), which extracts the model coefficients; ranef(), which extracts the BLUPs (and optional standard errors); and resid(), which extracts the residuals from the linear mixed sub-model. The intention of these functions is to have a common syntax with standard R packages for linear mixed models [26] and survival analysis [27]. Additionally, plotting capabilities are included in joineRML. These include trace plots for assessment of convergence of the MCEM algorithm, and caterpillar plots for subject-specific random effects (Table 2).

**Table 2:** Additional functions with descriptions that can be applied to objects of class mjoint^a^

Function(s)	Returns
logLik, AIC, BIC	the log-likelihood, Akaike information criterion and Bayesian information criterion statistics, respectively
coef, fixef	the fixed effects parameter estimates
ranef	the BLUPs (and optional standard errors)
print^a^, summary^c^	short and long model summary outputs, respectively
fitted, resid	the fitted values and raw residuals from the multivariate LMM sub-model, respectively
plot ^b^	the MCEM algorithm convergence trace plots
sigma	the residual standard errors from the LMM sub-model
vcov	the variance-covariance matrix of the main parameters of the fitted model (except the baseline hazard)
getVarCov	the random effects variance-covariance matrix
confint	the confidence intervals based on asymptotic normality
update	specific parts of a fitted model can be updated, e.g. by adding or removing terms from a sub-model, and then re-fitted
sampleData	sample data (with or without replacement) from a joint model

^a^print() also applies to objects of class summary.mjoint and bootSE inheriting from the summary() and bootSE() functions, respectively

^b^plot() also accepts objects of class ranef.mjoint inheriting from the ranef() function, which displays a caterpillar plot (with 95% prediction intervals) for each random effect

^c^summary() can also take the optional argument of an object of class bootSE inheriting from the function bootSE(), which overrides the approximate SEs and CIs with those from a bootstrap estimation routine

The package also provides several datasets, and a function simData() that allows for simulation of data from joint models with multiple longitudinal outcomes. joineRML can also fit univariate joint models, however in this case we would currently recommend that the R packages joineR [29], JM [28], or frailtypack [38] are used, which are optimized for the univariate case and exploits Gaussian quadrature. In addition, these packages allow for extensions to more complex cases; for example, competing risks [28, 29] and recurrent events [38].

## Results

### Simulation analysis

A simulation study was conducted assuming two longitudinal outcomes and *n*=200 subjects. Longitudinal data were simulated according to a follow-up schedule of 6 time points (at times 0,1,…,5), with each model including subject-and-outcome-specific random-intercepts and random-slopes: ***b***_*i*_=(*b*_0*i*1_,*b*_1*i*1_,*b*_0*i*2_,*b*_1*i*2_)^⊤^, Correlation was induced between the 2 outcomes by assuming correlation of − 0.5 between the random intercepts for each outcome. Event times were simulated from a Gompertz distribution with shape *θ*_1_=−3.5 and scale exp(*θ*_0_)= exp(0.25)≈1.28, following the methodology described by Austin [39]. Independent censoring times were drawn from an exponential distribution with rate 0.05. Any subject where the event and censoring time exceeded 5 was administratively censored at the truncation time *C*=5.1. For all sub-models, we included a pair of covariates ***X***_*i*_=(*x*_*i*1_,*x*_*i*2_)^⊤^, where *x*_*i*1_ is a continuous covariate independently drawn from *N*(0,1) and *x*_*i*2_ is a binary covariate independently drawn from *Bin*(1,0.5). The sub-models are given as

$$\begin{array}{@{}rcl@{}} y_{ijk} &=& (\beta_{0, k} + b_{i0k}) + (\beta_{1, k} + b_{i1k}) t_{j} \\&\quad+& \beta_{2, k} x_{i1} + \beta_{3, k} x_{i2} + \varepsilon_{ijk}, \text{ for } k = 1, 2; \\ \lambda_{i}(t) &=& \exp\left\{ (\theta_{0} + \theta_{1} t) + \gamma_{v1} x_{i1} + \gamma_{v2} x_{i2} \right.\\&\quad+&\left. \gamma_{y1} (b_{i01} + b_{i11} t) + \gamma_{y2} (b_{i02} + b_{i12} t) \right\}; \\ {b}_{i} &\sim& N_{4}(0, \boldsymbol{D}); \\ \varepsilon_{ijk} &\sim& N(0, \sigma_{k}^{2}), \end{array} $$

where *D* is specified unstructured (4×4)-covariance matrix with 10 unique parameters. Simulating datasets is straightforward using the joineRML package by means of the simData() function. The true parameter values and results from 500 simulations are shown in Table 3. In particular, we display the mean estimate, the bias, the empirical SE (= the standard deviation of the the parameter estimates); the mean SE (= the mean SE of each parameter calculated for each fitted model); the mean square error (MSE), and the coverage. The results confirm that the model fitting algorithm generally performs well.

**Table 3:** Results of simulation study

Parameter	True value	Mean estimated value	Empirical SE	Mean SE	Bias	MSE	Coverage
*D*_11_	0.2500	0.2411	0.0435	—	−0.0089	0.0020	—
*D*_21_	0.0000	0.0010	0.0136	—	0.0010	0.0002	—
*D*_31_	−0.1250	−0.1212	0.0295	—	0.0038	0.0009	—
*D*_41_	0.0000	−0.0006	0.0127	—	−0.0006	0.0002	—
*D*_22_	0.0400	0.0396	0.0072	—	−0.0004	0.0001	—
*D*_32_	0.0000	−0.0002	0.0138	—	−0.0002	0.0002	—
*D*_42_	0.0000	−0.0001	0.0055	—	−0.0001	0.0000	—
*D*_33_	0.2500	0.2420	0.0400	—	−0.0080	0.0017	—
*D*_43_	0.0000	0.0007	0.0134	—	0.0007	0.0002	—
*D*_44_	0.0400	0.0399	0.0075	—	−0.0001	0.0001	—
*β*_0,1_	0.0000	0.0028	0.0612	0.0660	0.0028	0.0038	0.9660
*β*_1,1_	1.0000	1.0012	0.0218	0.0229	0.0012	0.0005	0.9500
*β*_2,1_	1.0000	1.0010	0.0449	0.0470	0.0010	0.0020	0.9540
*β*_3,1_	1.0000	0.9932	0.0897	0.0925	−0.0068	0.0081	0.9440
$\sigma _{1}^{2}$	0.2500	0.2506	0.0165	0.0171	0.0006	0.0003	0.9560
*β*_0,2_	0.0000	−0.0026	0.0637	0.0655	−0.0026	0.0041	0.9660
*β*_1,2_	−1.0000	−1.0011	0.0229	0.0223	−0.0011	0.0005	0.9480
*β*_2,2_	0.0000	0.0008	0.0399	0.0472	0.0008	0.0016	0.9700
*β*_3,2_	0.5000	0.5061	0.0894	0.0923	0.0061	0.0080	0.9540
$\sigma _{2}^{2}$	0.2500	0.2501	0.0162	0.0171	0.0001	0.0003	0.9540
*γ*_*v*1_	0.0000	0.0011	0.1243	0.1392	0.0011	0.0155	0.9720
*γ*_*v*2_	1.0000	1.0487	0.2837	0.2750	0.0487	0.0829	0.9340
*γ*_*y*1_	−0.5000	−0.5121	0.1936	0.2084	−0.0121	0.0376	0.9560
*γ*_*y*2_	1.0000	1.0311	0.2220	0.2145	0.0311	0.0502	0.9400

A second simulation analysis was conducted using the parameters above (with *n*=100 subjects per dataset). However, in this case we used a heavier-tailed distribution for the random effects: a multivariate *t*_5_ distribution [40]. The bias for the fixed effect coefficients was comparable to the multivariate normal random effects simulation study (above). The empirical standard error was consistently smaller than the mean standard error, resulting in coverage between 95% and 99% for the coefficient parameters. Rizopoulos et al. [41] noted that the misspecification of the random effects distributions was minimised as the number of longitudinal measurements per subject increased, but that the standard errors are generally affected. These findings are broadly in agreement with the simulation study conducted here, and other studies [42, 43]. Choi et al. [44] provide a review of existing research on the misspecification of random effects in joint modelling.

### Example

We consider the primary biliary cirrhosis (PBC) data collected at the Mayo Clinic between 1974 to 1984 [45]. This dataset has been widely analyzed using joint modelling methods [18, 46, 47]. PBC is a long-term liver disease in which the bile ducts in the liver become damaged. Progressively, this leads to a build-up of bile in the liver, which can damage it and eventually lead to cirrhosis. If PBC is not treated or reaches an advanced stage, it can lead to several major complications, including mortality. In this study, 312 patients were randomised to receive D-penicillamine (*n*=158) or placebo (*n*=154). In this example we analyse the subset of patients randomized to placebo.

**Fig. 1:**
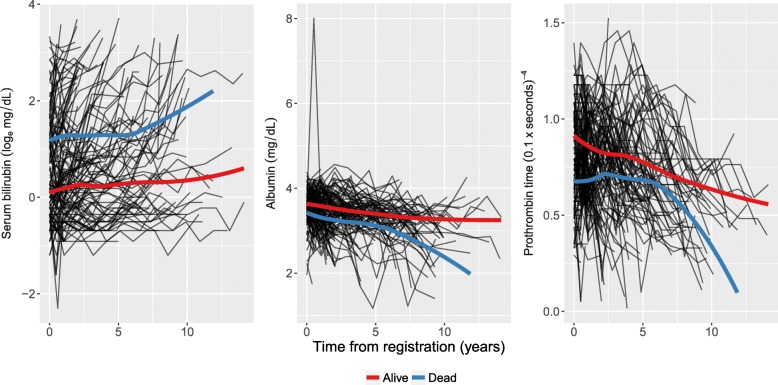
Longitudinal trajectory plots. The black lines show individual subject trajectories, and the coloured lines show smoothed (LOESS) curves stratified by whether the patient experienced the endpoint (blue) or not (red)

**Fig. 2:**
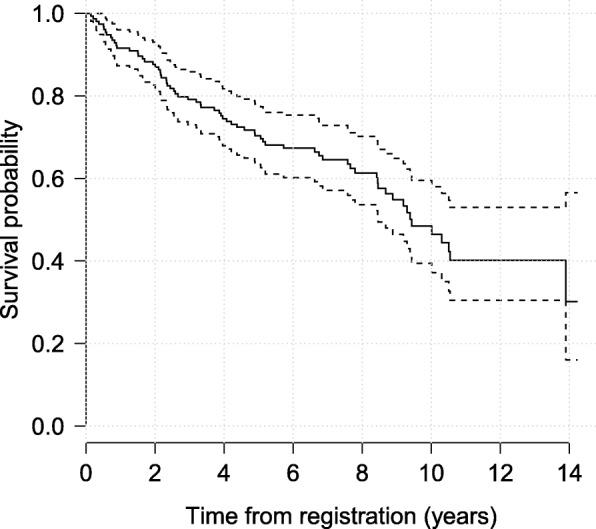
Kaplan-Meier curve for overall survival. A pointwise 95% band is shown (dashed lines). In total, 69 patients (of 154) died during follow-up

Patients with PBC typically have abnormalities in several blood tests; hence, during follow-up several biomarkers associated with liver function were serially recorded for these patients. We consider three biomarkers: serum bilirunbin (denoted serBilir in the model and data; measured in units of mg/dl), serum albumin (albumin; mg/dl), and prothrombin time (prothrombin; seconds). Patients had a mean 6.3 (SD = 3.7) visits (including baseline). The data can be accessed from the joineRML package via the command data(pbc2). Profile plots for each biomarker are shown in Fig. 1, indicating distinct differences in trajectories between the those who died during follow-up and those who did not (right-censored cases). A Kaplan-Meier curve for overall survival is shown in Fig. 2. There were a total of 69 (44.8%) deaths during follow-up in the placebo subset.

We fit a relatively simple joint model for the purposes of demonstration, which encompasses the following trivariate longitudinal data sub-model:

$$\begin{array}{@{}rcl@{}} {{}\selectfont{\begin{aligned} \log(\texttt{serBilir}) &\,=\, (\beta_{0, 1} \,+\, b_{0i, 1}) \,+\, (\beta_{1,1} \,+\, b_{1i, 1}) \texttt{year} \,+\, \varepsilon_{ij1}, \\ \texttt{albumin} &\,=\, (\beta_{0, 2} \,+\, b_{0i, 2}) \,+\, (\beta_{1,2}\! + \!b_{1i, 2}) \texttt{year} \,+\, \varepsilon_{ij2}, \\ (0.1 \times \texttt{prothrombin})^{-4} &\,=\, (\beta_{0, 3} \,+\, b_{0i, 3}) \,+\, (\beta_{1,3} \,+\, b_{1i, 3}) \texttt{year} \,+\, \varepsilon_{ij3}, \\ \boldsymbol{b}_{i} &\!\sim\! N_{6} (0, \boldsymbol{D}), \text{ and } \varepsilon_{ijk} \!\sim\! N(0, \sigma_{k}^{2}) \text{ for } k\,=\,1, \!2,\! 3; \end{aligned}}} \end{array} $$

and a time-to-event sub-model for the study endpoint of death:

$$\begin{array}{@{}rcl@{}} \lambda_{i}(t) &=& \lambda_{0}(t) \exp \left\{\gamma_{v} \texttt{age}_{i} + W_{2i}(t) \right\}, \\ W_{2i}(t) &=& \gamma_{\texttt{bil}}(b_{0i, 1} + b_{1i, 1} t) + \gamma_{\texttt{alb}}(b_{0i, 2} + b_{1i, 2} t) \\ &\quad+& \gamma_{\texttt{pro}}(b_{0i, 3} + b_{1i, 3} t). \end{array} $$

The log transformation of bilirubin is standard, and confirmed reasonable based on inspection of Q-Q plots for residuals from a separate fitted linear mixed model fitted using the lme() function from the R package nlme. Albumin did not require transformation. Residuals were grossly non-normal for prothrombin time using both untransformed and log-transformed outcomes. Therefore, a Box-Cox transformation was applied, which suggested an inverse-quartic transform might be suitable, which was confirmed by inspection of a Q-Q plot. The pairwise correlations for baseline measurements between the three transformed markers were 0.19 (prothrombin time *vs.* albumin), − 0.30 (bilirubin *vs.* prothrombin time and albumin). The model is fit using the joineRML R package (version 0.2.0) using the following code.


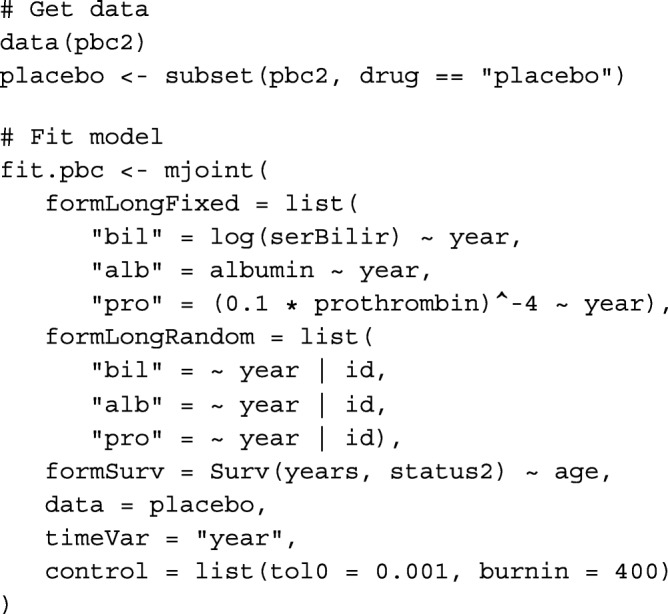


Here, we have specified a more stringent tolerance value for *ε*_0_ than the default setting in mjoint(). Additionally, the burn-in phase was increased to 400 iterations after inspection of convergence trace plots. The model fits in 3.1 min on a MacBook Air 1.6GHz Intel Core i5 with 8GB or RAM running R version 3.3.0, having completed 423 MCEM iterations (not including the EM algorithm iterations performed for determining the initial values of the separate multivariate linear mixed sub-model) with a final MC size of *M*=3528. The fitted model results are shown in Table 4.

**Table 4:** Fitted multivariate and separate univariate joint models to the PBC data

	joineRML (NR)			joineRML (GN)			Bootstrap		joineR		
	Estimate	SE	95% CI^d^	Estimate	SE	95% CI^d^	SE	95% CI^e^	Estimate	SE	95% CI^e^
*β*_0,1_	0.5541	0.0858	(0.3859, 0.7223)	0.5549	0.0846	(0.3892, 0.7207)	0.0800	(0.4264, 0.7435)	0.5545	0.0838	(0.3802, 0.7031)
*β*_1,1_	0.2009	0.0201	(0.1616, 0.2402)	0.2008	0.0201	(0.1614, 0.2402)	0.0204	(0.1669, 0.2468)	0.1808	0.0209	(0.1430, 0.2324)
*β*_0,2_	3.5549	0.0356	(3.4850, 3.6248)	3.5546	0.0357	(3.4846, 3.6245)	0.0255	(3.4972, 3.5904)	3.5437	0.0333	(3.4418, 3.6095)
*β*_1,2_	−0.1245	0.0101	(−0.1444, −0.1047)	−0.1246	0.0101	(−0.1444, −0.1047)	0.0120	(−0.1489, −0.1063)	−0.0997	0.0113	(−0.1256, −0.0773)
*β*_0,3_	0.8304	0.0212	(0.7888, 0.8719)	0.8301	0.0210	(0.7888, 0.8713)	0.0196	(0.7953, 0.8638)	0.8233	0.0220	(0.7818, 0.8677)
*β*_1,3_	−0.0577	0.0062	(−0.0699, −0.0456)	−0.0577	0.0062	(−0.0698, −0.0455)	0.0057	(−0.0698, −0.0486)	−0.0447	0.0052	(−0.0555, −0.0362)
*γ*_*v*_	0.0462	0.0151	(0.0166, 0.0759)	0.0462	0.0152	(0.0165, 0.0759)	0.0173	(0.0198, 0.0880)	0.0575^a^	0.0123^a^	(0.0314, 0.0760)^a^
									0.0413^b^	0.0150^b^	(0.0113, 0.0714)^b^
									0.0424^c^	0.0157^c^	(0.0146, 0.0724)^c^
*γ*_bil_	0.8181	0.2046	(0.4171, 1.2191)	0.8187	0.2036	(0.4197, 1.2177)	0.2153	(0.5172, 1.4021)	1.2182	0.1654	(0.9800, 1.5331)
*γ*_alb_	−1.7060	0.6181	(−2.9173, −0.4946)	−1.6973	0.6163	(−2.9053, −0.4893)	0.7562	(−3.3862, −0.5188)	−3.0770	0.6052	(−4.7133, −2.1987)
*γ*_pro_	−2.2085	1.6070	(−5.3582, 0.9412)	−2.2148	1.6133	(−5.3768, 0.9472)	1.6094	(−5.3050, 0.6723)	−7.2078	1.2640	(−10.5247, −5.2616)

Notation: SE = standard error; CI = confidence interval; NR = one-step Newton-Raphson update for ***γ***; GN = one-step Gauss-Newton-like update for ***γ***

^a^Separate model fit for serBilir

^b^Separate model fit for albumin

^c^Separate model fit for prothrombin

^d^SEs are calculated from the inverse profile empirical information matrix, and confidence intervals are based on normal approximations of the type $\hat {\boldsymbol {\theta }} \pm 1.96 \text {SE}(\hat {\boldsymbol {\theta }})$, where $\hat {\boldsymbol {\theta }}$ denote the estimated maximum likelihood estimates

^e^SEs and confidence intervals are derived from *B*=100 bootstrap samples, with confidence intervals based on the 2.5*%* and 97.5*%* percentiles. NB. one model failed to converge using joineRML within the maximum number of MC iterations, and so SEs and CIs are based on 99 bootstrap samples only

The fitted model indicated that an increase in the subject-specific random deviation from the population trajectory of serum bilirubin was significantly associated with increased hazard of death. A significant association was also detected for subject-specific decreases in albumin from the population mean trajectory. However, prothrombin time was not significantly associated with hazard of death, although its direction is clinically consistent with PBC disease. Albert and Shih [46] analysed the first 4-years follow-up from this dataset with the same 3 biomarkers and a discrete event time distribution using a regression calibration model. Their results were broadly consistent, although the effect of prothrombin time on the event time sub-model was strongly significant.

We also fitted 3 univariate joint models to each of the biomarkers and the event time sub-model using the R package joineR (version 1.2.0) owing to its optimization for such models. The LMM parameter estimates were similar, although the absolute magnitude of the slopes was smaller for the separate univariate models. Since 3 separate models were fitted, 3 estimates of *γ*_*v*_ were estimated, with the average comparable to the multivariate model estimate. The multivariate model estimates of ***γ***_*y*_=(*γ*_bil_,*γ*_alb_,*γ*_pro_)^⊤^ were substantially attenuated relative to the separate model estimates, although the directions remained consistent. It is also interesting to note that *γ*_pro_ was statistically significant in the univariate model. However, the univariate models are not accounting for the correlation between different outcomes, whereas the multivariate joint model does.

The model was refitted with the one-step Newton-Raphson update for *γ* replaced by a Gauss-Newton-like update in a time of 2.2 minutes for 419 MCEM iterations with a final MC size of *M*=6272. This is easily achieved by running the following code.


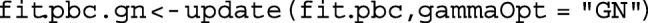


In addition, we bootstrapped this model with *B*=100 samples to estimate SEs and contrast them with the approximate estimates based on the inverse empirical profile information matrix. In practice, one should choose *B*>100, particularly if using bootstrap percentile confidence intervals; however, we used a small value to reduce the computational burden on this process. In a similar spirit, we relaxed the convergence criteria and reduced the number of burn-in iterations. This is easily implemented by running the following code, taking 1.8 h to fit.


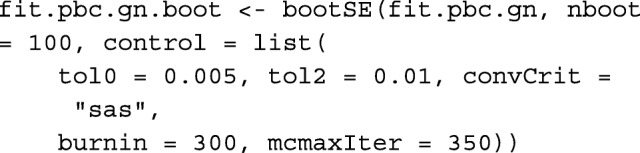


It was observed that the choice of gradient matrix in the *γ*-update led to virtually indistinguishable parameter estimates, although we note the same random seed was used in both cases. The bootstrap estimated SEs were broadly consistent with the approximate SEs, with no consistent pattern in underestimation observed.

## Discussion

Multivariate joint models introduce three types of correlations: (1) within-subject serial correlation for repeated measures; (2) between longitudinal outcomes correlation; and (3) correlation between the multivariate LMM and time-to-event sub-models. It is important to account for all of these types of correlations; however, some authors have reported collapsing their multivariate data to permit univariate joint models to be fitted. For example, Battes et al. [7] used an ad hoc approach of either summing or multiplying the three repeated continuous measures (standardized according to clinical upper reference limits of the biomarker assays), and then applying standard univariate joint models. Wang et al. [48] fitted separate univariate joint models to each longitudinal outcome in turn. Neither approach takes complete advantage of the correlation between the multiple longitudinal measures and the time-to-event outcome.

Here, we described a new R package joineRML that can fit the models described in this paper. This was demonstrated on a real-world dataset. Although in the fitted model we assumed linear trajectories for the biomarkers, splines could be straightforwardly employed, as have been used in other multivariate joint model applications [15], albeit at the cost of additional computational time. Despite a growing availability of software for univariate joint models, Hickey et al. [19] noted that there were very few options for fitting joint models involving multivariate longitudinal data. To the best of our knowledge, options are limited to the R packages JMbayes [49], rstanarm [50], and the Stata package stjm [47]. Moreover, none of these incorporates an unspecified baseline hazard. The first two packages use Markov chain Monte Carlo (MCMC) methods to fit the joint models. Bayesian models are potentially very useful for fitting joint models, and in particular for dynamic prediction; however, MCMC is also computationally demanding, especially in the case of multivariate models. Several other publications have made BUGS code available for use with WinBUGS and OpenBUGS (e.g. [51]), but these are not easily modifiable and post-fit computations are cumbersome.

joineRML is a new software package developed to fill a void in the joint modelling field, but is still in its infancy relative to highly developed univariate joint model packages such as the R package JM [28] and Stata package stjm [47]. Future developments of joineRML intend to cover several deficiencies. First, joineRML currently only permits an association structure of the form $W_{2i}(t) = \sum _{k=1}^{K} \gamma _{yk} W_{1i}^{(k)}(t)$. As has been demonstrated by others, the association might take different forms, including random-slopes and cumulative effects or some combination of multiple structures, and these may also be different for separate longitudinal outcomes [18]. Moreover, it is conceivable that separate longitudinal outcomes may interact in the hazard sub-model. Second, the use of MC integration provides a scalable solution to the issue of increasing dimensionality in the random effects. However, for simpler cases, e.g. bivariate models with random-intercepts and random-slopes (total of 4 random effects), Gaussian quadrature might be computationally superior; this trade-off requires further investigation. Third, joineRML can currently only model a single event time. However, there is a growing interest in competing risks [9] and recurrent events data [11], which if incorporated into joineRML, would provide a flexible all-round multivariate joint modelling platform. Competing risks [28, 29] and recurrent events [38] have been incorporated into joint modelling R packages already, but are limited to the case of a solitary longitudinal outcome. Of note, the PBC trial dataset analysed in this study includes times to the competing risk of liver transplantation. Fourth, with ever-increasing volumes of data collected during routine clinical visits, the need for software to fit joint models with very many longitudinal outcomes is foreseeable [52]. This would likely require the use of approximate methods for the numerical integration or data reduction methods. Fifth, additional residual diagnostics are necessary for assessing possible violations of model assumptions. The joineRML package has a resid() function for extracting the longitudinal sub-model residuals; however, these are complex for diagnostic purposes due to the informative dropout, hence the development of multiple-imputation based residuals [53].

## Conclusions

In this paper we have presented an extension of the classical joint model proposed by Henderson et al. [3] and an estimation procedure for fitting the models that builds on the foundations laid by Lin et al. [20]. In addition, we described a new R package joineRML that can fit the models described in this paper, which leverages the MCEM algorithm and which should scale well for increasing number of longitudinal outcomes. This software is timely, as it has previously been highlighted that there is a paucity of software available to fit such models [19]. The software is being regularly updated and improved.

## Availability and requirements

**Project name:**joineRML**Project home page:**https://github.com/graemeleehickey/joineRML/**Operating system(s):** platform independent **Programming language:** R **Other requirements:** none **License:** GNU GPL-3 Any restrictions to use by non-academics: none

## Additional file

Additional file 1 An appendix (appendix.pdf) is available that includes details on the score vector and M-step estimators. (PDF 220 kb)

## Abbreviations

BLUP: Best linear unbiased prediction
CRAN: The Comprehensive R Archive Network
EM: Expectation maximisation
LMM: Linear mixed models
MC: Monte Carlo
MCEM: Monte Carlo expectation maximisation
MLE: Maximum likelihood estimate
PBC: Primary biliary cirrhosis
SD: Standard deviation
SE: Standard error
