# Supplementary material for: joineRML: a joint model and software package for time-to-event and multivariate longitudinal outcomes
Source: BMC Med Res Methodol. 2018 Jun 7;18:50. doi: 10.1186/s12874-018-0502-1 (PMC6047371; doi:10.1186/s12874-018-0502-1)
Supplement: Supplementary file 1 — An appendix (appendix.pdf) is available that includes details on the score vector and M-step estimators. (PDF 220 kb) [file 12874_2018_502_MOESM1_ESM.pdf]

Appendix for "`joineRML`: A joint model and software package for time-to-event and multivariate longitudinal outcomes"

G. L. Hickey[1] & P. Philipson[2], A. L. Jorgensen[1], R. Kolamunnage-Dona[1]

[1]*Department of Biostatistics, Institute of Translational Medicine, University of Liverpool*
[2]*Department of Mathematics, Physics and Electrical Engineering, Northumbria University*

July 31, 2017

1 Likelihood

The *observed* data likelihood is given by

$$\prod_{i=1}^{n} \left(\int_{-\infty}^{\infty} f(\boldsymbol{y}_i \,|\, \boldsymbol{b}_i, \boldsymbol{\theta}) f(T_i, \delta_i \,|\, \boldsymbol{b}_i, \boldsymbol{\theta}) f(\boldsymbol{b}_i \,|\, \boldsymbol{\theta}) d\boldsymbol{b}_i \right), \tag{1}$$

where $\boldsymbol{\theta} = (\boldsymbol{\beta}^\top, \text{vech}(\boldsymbol{D}), \sigma_1^2, \dots, \sigma_K^2, \lambda_0(t), \boldsymbol{\gamma}_v^\top, \boldsymbol{\gamma}_y^\top)$ is the collection of unknown parameters that we want to estimate, with $\text{vech}(\boldsymbol{D})$ denoting the half-vectorisation operator that returns the vector of lower-triangular elements of matrix $\boldsymbol{D}$, and

$$\begin{aligned}
f(\boldsymbol{y}_i \,|\, \boldsymbol{b}_i, \boldsymbol{\theta}) &= \left(\prod_{k=1}^{K} (2\pi)^{-\frac{n_{ik}}{2}} \right) |\boldsymbol{\Sigma}_i|^{-\frac{1}{2}} \exp\left\{ -\frac{1}{2} (\boldsymbol{y}_i - \boldsymbol{X}_i\boldsymbol{\beta} - \boldsymbol{Z}_i\boldsymbol{b}_i)^\top \boldsymbol{\Sigma}_i^{-1} (\boldsymbol{y}_i - \boldsymbol{X}_i\boldsymbol{\beta} - \boldsymbol{Z}_i\boldsymbol{b}_i) \right\}, \\
f(T_i, \delta_i \,|\, \boldsymbol{b}_i; \boldsymbol{\theta}) &= \left[\lambda_0(T_i) \exp\left\{ \boldsymbol{v}_i^\top \gamma_v + W_{2i}(T_i, \boldsymbol{b}_i) \right\} \right]^{\delta_i} \exp\left\{ -\int_0^{T_i} \lambda_0(u) \exp\left\{ \boldsymbol{v}_i^\top \gamma_v + W_{2i}(u, \boldsymbol{b}_i) \right\} du \right\}, \\
f(\boldsymbol{b}_i \,|\, \boldsymbol{\theta}) &= (2\pi)^{-\frac{r}{2}} |\boldsymbol{D}|^{-\frac{1}{2}} \exp\left\{ -\frac{1}{2} \boldsymbol{b}_i^\top \boldsymbol{D}^{-1} \boldsymbol{b}_i \right\},
\end{aligned}$$

where $r = \sum_{k=1}^{K} r_k$ is the total dimensionality of the random effects variance-covariance matrix.

2 Score & update equations

From (1), the expected complete-data log-likelihood is given by

$$Q(\boldsymbol{\theta} \,|\, \hat{\boldsymbol{\theta}}^{(m)}) = \sum_{i=1}^{n} \int_{-\infty}^{\infty} \left\{ \log f(\boldsymbol{y}_i, T_i, \delta_i, \boldsymbol{b}_i \,|\, \boldsymbol{\theta}) \right\} f(\boldsymbol{b}_i \,|\, T_i, \delta_i, \boldsymbol{y}_i, \hat{\boldsymbol{\theta}}^{(m)}) d\boldsymbol{b}_i$$

where the expectation is taken over the conditional random effects distribution $f(\boldsymbol{b}_i \,|\, T_i, \delta_i, \boldsymbol{y}_i, \hat{\boldsymbol{\theta}}^{(m)})$. Hence, the updates require expectations about the random effects be calculated of the form $\mathbb{E}\left[h(\boldsymbol{b}_i) \,|\, T_i, \delta_i, \boldsymbol{y}_i; \hat{\boldsymbol{\theta}}^{(m)} \right]$, which, in the interests of brevity, we denote here onwards as $\mathbb{E}\left[h(\boldsymbol{b}_i) \right]$ in the update estimators. This expectation is conditional on the observed data $(T_i, \delta_i, \boldsymbol{y}_i)$ for each subject, the covariates (including measurement

times) $(\boldsymbol{X}_i, \boldsymbol{Z}_i, \boldsymbol{v}_i)$, which are implicitly dependent, and the current estimate of the model parameters $\boldsymbol{\theta}$ from the m-th iteration.

We can decompose the complete-data log-likelihood for subject i into

$$\log f(\boldsymbol{y}_i, T_i, \delta_i, \boldsymbol{b}_i \mid \boldsymbol{\theta}) = \log f(\boldsymbol{y}_i \mid \boldsymbol{b}_i, \boldsymbol{\theta}) + \log f(T_i, \delta_i \mid \boldsymbol{b}_i, \boldsymbol{\theta}) + \log f(\boldsymbol{b}_i \mid \boldsymbol{\theta}),$$

where

$$\begin{aligned} \log f(\boldsymbol{y}_i \mid \boldsymbol{b}_i, \boldsymbol{\theta}) &= -\frac{1}{2}\left\{\left(\sum_{k=1}^{K} n_{ik}\right)\log(2\pi) + \log|\boldsymbol{\Sigma}_i| + (\boldsymbol{y}_i - \boldsymbol{X}_i\boldsymbol{\beta} - \boldsymbol{Z}_i\boldsymbol{b}_i)^\top \boldsymbol{\Sigma}_i^{-1}(\boldsymbol{y}_i - \boldsymbol{X}_i\boldsymbol{\beta} - \boldsymbol{Z}_i\boldsymbol{b}_i)\right\} \quad (2) \\ \log f(T_i, \delta_i \mid \boldsymbol{b}_i, \boldsymbol{\theta}) &= \delta_i \log\lambda_0(T_i) + \delta_i\left[\boldsymbol{v}_i^\top\boldsymbol{\gamma}_v + W_{2i}(T_i, \boldsymbol{b}_i)\right] - \int_0^{T_i} \lambda_0(u)\exp\left\{\boldsymbol{v}_i^\top\boldsymbol{\gamma}_v + W_{2i}(u, \boldsymbol{b}_i)\right\} du, \\ \log f(\boldsymbol{b}_i \mid \boldsymbol{\theta}) &= -\frac{1}{2}\left\{r\log(2\pi) + \log|\boldsymbol{D}| + \boldsymbol{b}_i^\top \boldsymbol{D}^{-1}\boldsymbol{b}_i\right\}. \end{aligned}$$

The update equations are then calculated from solving the score equations, $\partial Q(\boldsymbol{\theta} \mid \hat{\boldsymbol{\theta}}^{(m)})/\partial\boldsymbol{\theta}$, for $\boldsymbol{\theta}$. The components of the score vector are effectively given by Lin et al. [1]; however, there the random effects were hierarchically centred about the corresponding fixed effect terms as part of a current values parametrization, as well as being embedded in a frailty Cox model, which has consequences on the score here. The components of the score vector and corresponding M-step update equations for each parameter are given as follows.

2.1 $\lambda_0(t)$

The score with respective to $\lambda_0(t)$ is calculated as

$$S(\lambda_0(t)) = \sum_{i=1}^{n}\left\{\frac{\delta_i I(T_i = t)}{\lambda_0(t)} - \mathbb{E}\left[\exp\{\boldsymbol{v}_i^\top\boldsymbol{\gamma}_v + W_{2i}(t, \boldsymbol{b}_i)\}\right] I(T_i \geq t)\right\},$$

which leads to the closed-form update:

$$\hat{\lambda}_0(t) = \frac{\sum_{i=1}^{n}\delta_i I(T_i = t)}{\sum_{i=1}^{n}\mathbb{E}\left[\exp\left\{\boldsymbol{v}_i^\top\boldsymbol{\gamma}_v + W_{2i}(t, \boldsymbol{b}_i)\right\}\right] I(T_i \geq t)}, \quad (3)$$

which is only evaluated at distinct observed event times, t_j $(j = 1, \ldots, J)$, where $I(\mathcal{A})$ denotes an indicator function that takes the value 1 if event $\mathcal{A}$ occurs, and zero otherwise.

2.2 $\boldsymbol{\beta}$

The score with respect to $\boldsymbol{\beta}$ is calculated as

$$S(\boldsymbol{\beta}) = \sum_{i=1}^{n}\left\{\boldsymbol{X}_i^\top\boldsymbol{\Sigma}_i^{-1}(\boldsymbol{y}_i - \boldsymbol{X}_i\boldsymbol{\beta} - \boldsymbol{Z}_i\mathbb{E}[\boldsymbol{b}_i])\right\},$$

which leads to the closed-form update equation:

$$\begin{aligned}
\hat{\boldsymbol{\beta}} &= \left(\sum_{i=1}^{n} \boldsymbol{X}_i^\top \boldsymbol{\Sigma}_i^{-1} \boldsymbol{X}_i\right)^{-1} \left(\sum_{i=1}^{n} \boldsymbol{X}_i^\top \boldsymbol{\Sigma}_i^{-1} (\boldsymbol{y}_i - \boldsymbol{Z}_i \mathbb{E}[\boldsymbol{b}_i])\right), \\
&= \left(\sum_{i=1}^{n} \boldsymbol{X}_i^\top \boldsymbol{X}_i\right)^{-1} \left(\sum_{i=1}^{n} \boldsymbol{X}_i^\top (\boldsymbol{y}_i - \boldsymbol{Z}_i \mathbb{E}[\boldsymbol{b}_i])\right).
\end{aligned}$$

2.3 σ_k^2

Rewriting (2) as $\sum_{k=1}^{K} \log\{f(\boldsymbol{y}_{ik} \,|\, \boldsymbol{b}_{ik}, \boldsymbol{\theta})\}$, the score with respective to σ_k^2 is calculated as

$$\begin{aligned}
S(\sigma_k^2) &= -\frac{1}{2\sigma_k^2} \sum_{i=1}^{n} \left\{ n_{ik} - \frac{1}{\sigma_k^2} \mathbb{E}\left[(\boldsymbol{y}_{ik} - \boldsymbol{X}_{ik}\boldsymbol{\beta}_k - \boldsymbol{Z}_{ik}\boldsymbol{b}_{ik})^\top (\boldsymbol{y}_{ik} - \boldsymbol{X}_{ik}\boldsymbol{\beta}_k - \boldsymbol{Z}_{ik}\boldsymbol{b}_{ik}) \right] \right\} \\
&= -\frac{1}{2\sigma_k^2} \sum_{i=1}^{n} \left\{ n_{ik} - \frac{1}{\sigma_k^2} \left[(\boldsymbol{y}_{ik} - \boldsymbol{X}_{ik}\boldsymbol{\beta}_k)^\top (\boldsymbol{y}_{ik} - \boldsymbol{X}_{ik}\boldsymbol{\beta}_k - 2\boldsymbol{Z}_{ik}\mathbb{E}[\boldsymbol{b}_{ik}]) \right.\right. \\
&\qquad \left.\left. + \mathrm{trace}\left(\boldsymbol{Z}_{ik}^\top \boldsymbol{Z}_{ik} \mathbb{E}[\boldsymbol{b}_{ik}\boldsymbol{b}_{ik}^\top] \right) \right] \right\},
\end{aligned}$$

which leads to the closed-form update equation:

$$\begin{aligned}
\hat{\sigma}_k^2 &= \frac{1}{\sum_{i=1}^{n} n_{ik}} \sum_{i=1}^{n} \mathbb{E}\left\{ (\boldsymbol{y}_{ik} - \boldsymbol{X}_{ik}\boldsymbol{\beta}_k - \boldsymbol{Z}_{ik}\boldsymbol{b}_{ik})^\top (\boldsymbol{y}_{ik} - \boldsymbol{X}_{ik}\boldsymbol{\beta}_k - \boldsymbol{Z}_{ik}\boldsymbol{b}_{ik}) \right\} \\
&= \frac{1}{\sum_{i=1}^{n} n_{ik}} \sum_{i=1}^{n} \left\{ (\boldsymbol{y}_{ik} - \boldsymbol{X}_{ik}\boldsymbol{\beta}_k)^\top (\boldsymbol{y}_{ik} - \boldsymbol{X}_{ik}\boldsymbol{\beta}_k - 2\boldsymbol{Z}_{ik}\mathbb{E}[\boldsymbol{b}_{ik}]) + \mathrm{trace}\left(\boldsymbol{Z}_{ik}^\top \boldsymbol{Z}_{ik} \mathbb{E}[\boldsymbol{b}_{ik}\boldsymbol{b}_{ik}^\top] \right) \right\}.
\end{aligned}$$

2.4 $\boldsymbol{D}$

Using the transformation $\boldsymbol{V} = \boldsymbol{D}^{-1}$, the score with respect to V is calculated as

$$S(\boldsymbol{V}) = \frac{n}{2}\left\{ 2\boldsymbol{V}^{-1} - \mathrm{diag}(\boldsymbol{V}^{-1}) \right\} - \frac{1}{2}\left[2\sum_{i=1}^{n} \mathbb{E}\left[\boldsymbol{b}_i\boldsymbol{b}_i^\top\right] - \mathrm{diag}\left(\sum_{i=1}^{n} \mathbb{E}\left[\boldsymbol{b}_i\boldsymbol{b}_i^\top\right] \right) \right],$$

which leads to the closed-form update equation for $\boldsymbol{D}$:

$$\hat{\boldsymbol{D}} = \frac{1}{n}\sum_{i=1}^{n} \mathbb{E}\left[\boldsymbol{b}_i\boldsymbol{b}_i^\top\right].$$

We also require the score for $\boldsymbol{\theta}_b \equiv \mathrm{vech}(\boldsymbol{D})$, which can be calculated as

$$S(\boldsymbol{\theta}_b) = -\frac{n}{2}\mathrm{trace}\left(\boldsymbol{D}^{-1}\frac{\partial \boldsymbol{D}}{\partial \boldsymbol{\theta}_b} \right) + \frac{1}{2}\sum_{i=1}^{n} \left\{ \mathrm{trace}\left(\boldsymbol{D}^{-1}\frac{\partial \boldsymbol{D}}{\partial \boldsymbol{\theta}_b}\boldsymbol{D}^{-1}\mathbb{E}(\boldsymbol{b}_i\boldsymbol{b}_i^\top) \right) \right\}$$

2.5 $\boldsymbol{\gamma}$

The scores with respect to $\boldsymbol{\gamma}_v$ and $\boldsymbol{\gamma}_y$ do not have closed-form solutions. Therefore, they are updated jointly using a one-step Newton-Raphson algorithm iteration. We can write the scores with respect to $\boldsymbol{\gamma} = \left(\boldsymbol{\gamma}_v^\top, \boldsymbol{\gamma}_y^\top\right)^\top$

as

$$\begin{aligned} S(\boldsymbol{\gamma}) &= \sum_{i=1}^{n} \left[\delta_i \mathbb{E}\left[\tilde{\boldsymbol{v}}_i(T_i)\right] - \int_0^{T_i} \lambda_0(u) \mathbb{E}\left[\tilde{\boldsymbol{v}}_i(u) \exp\{\tilde{\boldsymbol{v}}_i^\top(u)\boldsymbol{\gamma}\}\right] du \right] \\ &= \sum_{i=1}^{n} \left[\delta_i \mathbb{E}\left[\tilde{\boldsymbol{v}}_i(T_i)\right] - \sum_{j=1}^{J} \lambda_0(t_j) \mathbb{E}\left[\tilde{\boldsymbol{v}}_i(t_j) \exp\{\tilde{\boldsymbol{v}}_i(t_j)^\top \boldsymbol{\gamma}\}\right] I(T_i \geq t_j) \right], \end{aligned}$$

where $\tilde{\boldsymbol{v}}_i(t) = \left(\boldsymbol{v}_i^\top, \boldsymbol{z}_{i1}^\top(t)\boldsymbol{b}_{i1}, \dots, \boldsymbol{z}_{iK}^\top(t)\boldsymbol{b}_{iK}\right)$ is a $(q+K)$-vector, and the integration over the survival process has been replaced with a finite summation over the process evaluated at the unique failure times, since the non-parametric estimator of baseline hazard is zero except at observed failure times [2]. As $\lambda_0(t_j)$ is a function of $\boldsymbol{\gamma}$, this is not a closed-form solution. Substituting $\lambda_0(t)$ by $\hat{\lambda}_0(t)$ from (3), which is a function of $\boldsymbol{\gamma}$ and the observed data itself, gives a score that is independent of $\lambda_0(t)$. Discussion of this in the context of univariate joint modelling is given by Hsieh et al. [3]. A useful result is that the maximum profile likelihood estimator is the same as the maximum partial likelihood estimator [4], meaning that plugging-in the estimator $\hat{\lambda}_0(t)$ into (1) gives a profile likelihood independent of $\lambda_0(t)$.

The information for $\boldsymbol{\gamma}$ is calculated by taking the partial derivative of the score above, and is given by

$$I(\boldsymbol{\gamma}) \equiv -\frac{\partial}{\partial \boldsymbol{\gamma}} S(\boldsymbol{\gamma}) = \sum_{i=1}^{n}\sum_{j=1}^{J} \left\{ \hat{\lambda}_0(t_j) I(T_i \geq t_j) \mathbb{E}\left[\tilde{\boldsymbol{v}}_i^{\otimes 2}(t_j) \exp\{\tilde{\boldsymbol{v}}_i^\top(t_j)\boldsymbol{\gamma}\}\right] \right\} - \sum_{j=1}^{J} \frac{\hat{\lambda}_0(t_j)^2 \boldsymbol{\Gamma}(t_j)}{\sum_{i=1}^{n} \delta_i I(T_i = t_j)}.$$

where

$$\boldsymbol{\Gamma}(t_j) = \left\{ \sum_{i=1}^{n} \mathbb{E}\left[\tilde{\boldsymbol{v}}_i(t_j) \exp\{\tilde{\boldsymbol{v}}_i^\top(t_j)\boldsymbol{\gamma}\}\right] I(T_i \geq t_j) \right\}^{\otimes 2},$$

$\hat{\lambda}_0(t)$ is given by (3), which is also a function of $\boldsymbol{\gamma}$, and $\boldsymbol{a}^{\otimes 2} = \boldsymbol{a}\boldsymbol{a}^\top$ is the outer-product of the vector $\boldsymbol{a}$. In practice, calculation of $I(\boldsymbol{\gamma})$ is computationally expensive to evaluate. Therefore, in some situations we may want to approximate it. One approximation we consider is a Gauss-Newton-like approximation [5, p. 8], which exploits the empirical information matrix approach calculation, but restricted to $\boldsymbol{\gamma}$ only. To further compensate for this approximation, we also use a nominal step-size of 0.5 rather than 1, which is used when exactly calculating $I(\boldsymbol{\gamma})$. Hence, the one-step block update at the $(m+1)$-th EM algorithm iteration is

$$\hat{\boldsymbol{\gamma}}^{(m+1)} = \hat{\boldsymbol{\gamma}}^{(m)} + I\left(\hat{\boldsymbol{\gamma}}^{(m)}\right)^{-1} S\left(\hat{\boldsymbol{\gamma}}^{(m)}\right).$$

References

[1] H Lin, C. E. McCulloch, and S. T. Mayne. “Maximum likelihood estimation in the joint analysis of time-to-event and multiple longitudinal variables”. In: *Statistics in Medicine* 21 (2002), pp. 2369–2382.

[2] R Henderson, P. J. Diggle, and A Dobson. “Joint modelling of longitudinal measurements and event time data”. In: *Biostatistics* 1.4 (2000), pp. 465–480.

[3] F Hsieh, Y. K. Tseng, and J. L. Wang. “Joint modeling of survival and longitudinal data: Likelihood approach revisited”. In: *Biometrics* 62.4 (2006), pp. 1037–1043.

[4] H Lin. “A finite mixture model for joint longitudinal biomarker and survival outcome responses”. Ph.D. Cornell University, 2000.

[5] G. J. McLachlan and T Krishnan. *The EM Algorithm and Extensions*. Second. Wiley-Interscience, 2008.
